# Quality of life and its selected determinants in the group of patients with surgically treated spinal tumors

**DOI:** 10.3389/fonc.2023.1213258

**Published:** 2023-11-02

**Authors:** Beata Barańska, Dariusz Bazaliński, Grzegorz Guzik, Maria Kózka, Robert Ślusarz, Paweł Więch

**Affiliations:** ^1^ Podkarpackie Specialist Oncology Centre, Specialist Hospital in Brzozów, Brzozów, Poland; ^2^ Institute of Health Sciences, College of Medical Sciences, University of Rzeszów, Rzeszów, Poland; ^3^ Institute of Health Protection, State University of Applied Sciences in Przemyśl, Przemyśl, Poland; ^4^ Faculty of Health Sciences, Institute of Nursing and Midwifery, Department of Clinical Nursing, Jagiellonian University Medical College, Krakow, Poland; ^5^ Neurological and Neurosurgical Nursing Department, Faculty of Health Science, Collegium Medicum in Bydgoszcz, Nicolaus Copernicus University in Toruń, Toruń, Poland

**Keywords:** bone metastases, quality of life, disease acceptance, self-care, spinal tumors

## Abstract

**Introduction:**

Spinal metastases are a common manifestation of advanced neoplastic disease. Destructive neoplastic lesions within the axial skeleton cause unrelieved pain and nervous system disorders involving spinal stenosis and other neural structures. The development of new systemic therapies, radiotherapy and minimally invasive spinal surgeries has increased patients’ quality of life by minimising pain and neurological disorders due to vertebral neoplastic infiltration. The aim of the study was to assess the patients’ quality of life before and after spine stabilisation surgery with spinal cord decompression to relieve the pressure associated with neoplastic destruction.

**Materials and Methods:**

The study involved 115 subjects with spinal metastases in the preoperative period and 3–4 months after the surgery based on the inclusion criteria (metastatic spinal tumour, sensorimotor dysfunction). The data were collected using the following tools: the Rotterdam Symptom Checklist (RSCL-Rotterdam Symptom Checklist), Acceptance Illness Scale (AIS scale), Activities of Daily Living Scale (ADL scale) and Visual Analogue Scale (VAS). The correlation coefficient was calculated using Spearman’s rho assuming the significance level at α = 0.05 (p<0.05).

**Results:**

A higher quality of life was found after surgery (p<0.001) in terms of experiencing physical symptoms (30.7 ± 11.96 points before surgery vs. 20.91 ± 13.00 points after surgery) and psychological symptoms (43.98 ± 14.82 points before surgery vs. 31.35 ± 14.86 points after surgery). The activity level of the subjects also improved (p<0.001; 36.56 ± 22.43 points to 43.55 ± 20.40 points). The level of disease acceptance in the study group was higher after the surgery compared to the preoperative assessment. The subjects with a high level of disease acceptance presented a higher quality of life postoperatively. The independence of the subjects in performing everyday activities after the operation influenced the quality of life, in terms of somatic symptoms (p=0.006), mental symptoms (p=0.001) and activity (p<0.001). Along with the improvement in functional capacity, the quality of life in terms of symptoms and activity levels increased.

**Conclusion:**

The study showed that spinal cord decompression surgery improves the quality of life of patients by reducing neurological dysfunction, increasing the acceptance of the disease and the ability to perform activities of daily living (ADL). Sociodemographic variables did not affect the quality of life of the respondents.

## 1 Introduction

Modern advances in medical knowledge, early diagnostics, access to screening, progress in pharmacology and radiotherapy offer a chance to cure or significantly extend the lifespan of people with neoplastic disease (1). Despite expert observations and epidemiological data, many patients are still diagnosed in the advanced stages of disease and will require professional care and palliative treatment. Global data indicate that approximately 30 million patients (3% of the worldwide population) need holistic palliative care (2–4). The morphology of the metastatic lesions varies. The most common are osteolytic (70%), mixed (20%) and osteoblastic (10%) metastases. They may occur at any stage of the neoplastic disease and most often affect patients with breast cancer (65–75%), prostate cancer (65–90%) and lung cancer (17–64%), and less often thyroid cancer (65%), bladder cancer and melanoma (14–45%) (5–7). The axial skeleton is the most common site of skeletal metastases with the thoracolumbar (70%) and lumbosacral (20%) sections being the most frequent and the cervical spine (10%) being the rarest location. Advances in standards of care and targeted systemic therapies constituted a substantial increase in life expectancy, which in turn led to an increasing incidence. Metastatic epidural spinal cord compression (MESCC) is a common debilitating complication occurring in 5–14% of patients, resulting from clinically advanced cancer. The rapidly progressing symptoms of MESCC require immediate treatment. Standard treatment protocols are based on corticosteroid therapy, radiotherapy and surgical methods of spinal cord decompression (8). The role of surgery in recent years has significantly increased compared to other methods. Patients may benefit from surgical decompression followed by radiotherapy in terms of functional capacity, pain relief and life expectancy (9).

Bone metastases dramatically reduce the patients’ quality of life. Infiltration or compression of the spinal cord predispose to neurological dysfunction. Severe pain, pathological fractures, paresis and life-threatening hypercalcemia are the most common symptoms requiring radiotherapy or surgery (10, 11). Surgical management has a palliative character; it is intended to improve the patient’s ability to function with advanced neoplastic disease. The aim of the study was to assess patients’ quality of life before and after spinal surgery for decompression of the nerve structures.

## 2 Materials and methods

### 2.1 Ethics

The study protocol was approved by the ethics committees of the involved institution (Bioethics Commission at the University of Rzeszow: Resolution no. 2016/12/7 on 01 December 2016). Moreover, the guidelines of the Declaration of Helsinki were followed during the course of the conducted research. The participants were informed of the purpose of the study and could withdraw at any time without giving any reason.

### 2.2 Subjects

**Figure 1:**
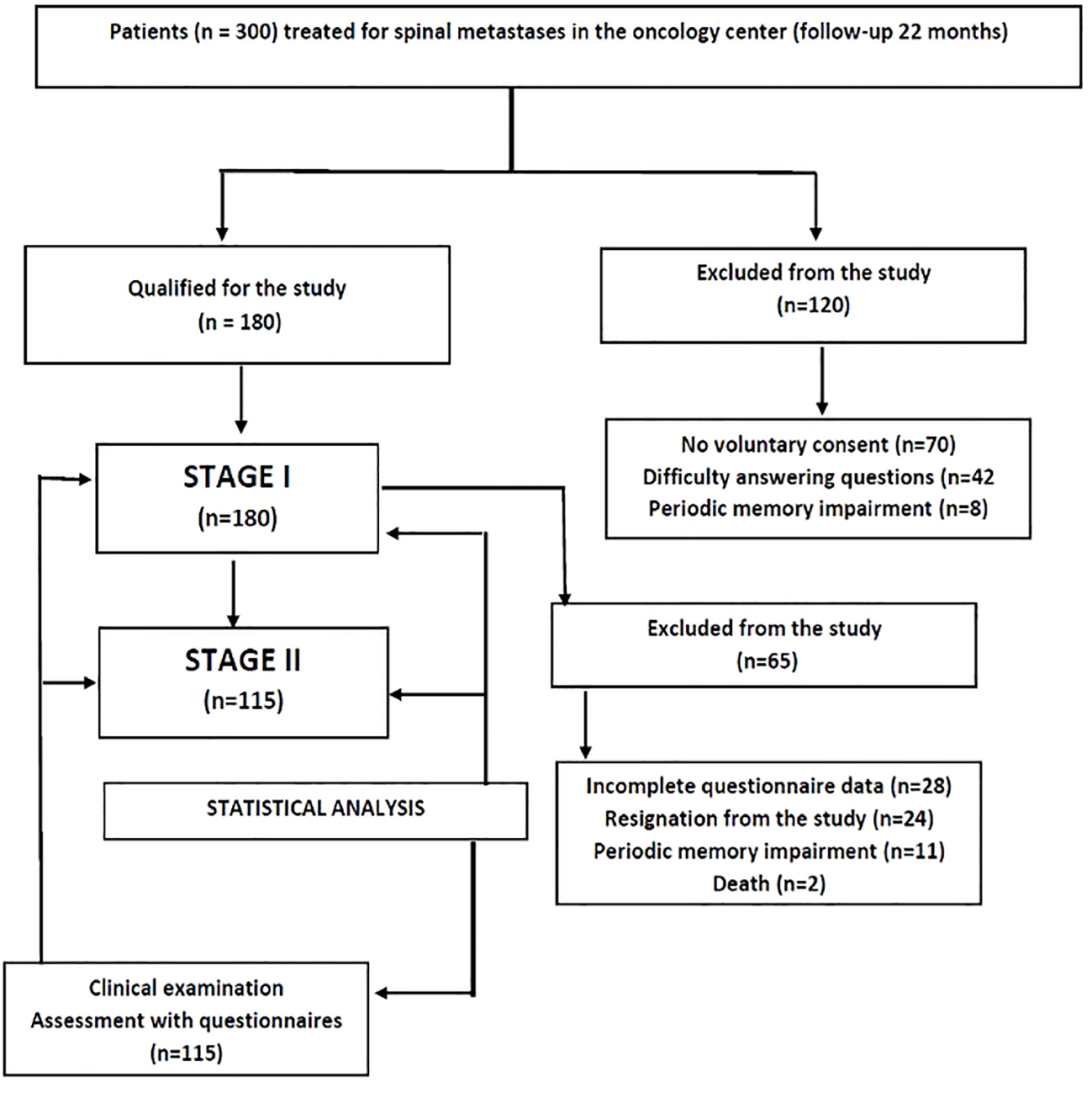
Flow chart demonstrating study participants selection.

A prospective single-centre study was conducted based on the method of estimation and diagnostic survey. Out of 300 patients treated surgically for skeletal metastases in 2018–2019 at a regional oncology centre (Podkarpackie Oncology Centre), 115 people who met the inclusion criteria (spinal metastasis and sensorimotor dysfunction) qualified for the study. A prospective, single-centre study was conducted based on the estimation method and a diagnostic survey. MESCC was defined radiologically (CT, MRI) as actual displacement of the spinal cord (through the epidural mass) from its physiological position in the spinal canal. Patients must also have had at least one neurological symptom (including pain) and not had paraplegia for more than 48 hours prior to study entry (according to ASIA). Surgery began within a median time of 16 h (interquartile range 10–22h admission to surgical incision). The MESCC had to be confined to a single area, which could include several adjacent spinal segments or vertebrae. Inclusion criteria for the main study included status after spinal surgery (laminectomy with posterior stabilization (n=78), corpectomy (n=37) with implantation of the shaft prosthesis) due to metastasis of a malignant tumour originally located elsewhere, voluntary consent to participate in the study and self-completed tools. The assessment of the condition of the patients was made up to 48 hours after admission and repeated 3-4 months after the operation (**Figure 1**). The study group accounted for 38.33% of all patients with spinal metastases treated in the Podkarpackie Oncology Centre during the study period.

### 2.3 Assessments and research tools

To collect the data, the patient’s medical records and standardised tools were used. Data on the clinical and functional status were obtained from the medical records (comorbidities, clinical diagnosis, location of metastatic lesions in the spine and indications for orthopaedic treatment) and clinical examination. The Rotterdam Symptom Check List (RSCL) was used to assess the overall quality of life of the respondents as well as their physical, mental and activity levels (12). The disease acceptance level was assessed using the Acceptance Illness Scale (AIS) (13, 14). Daily activities were assessed using Activities of Daily Living scale (ADL) (15). The pain level was assessed using the Visual Analogue Scale (VAS) (16). Neurological status was assessed using the American Spinal Injury Association scale (ASIA scale) (17).

### 2.4 Statistical analysis

The statistical analysis was performed using the STATISTICA 13 software. Quantitative variables were presented in the form of the arithmetic mean, standard deviation, minimum, maximum and median. Qualitative variables were presented as number and percentage. The differences between the variables were verified with the Mann-Whitney test and the Kruskal-Wallis test (for independent variables), the Wilcoxon signed-rank test or the McNemar-Bowker test (for dependent variables) and by calculating the Spearman’s rho correlation coefficient. The choice of tests was dictated by the lack of normality of the distribution of variables (verified with the Kolmogorov-Smirnov and Shapiro-Wilk test) or the lack of equipotency of the studied groups (verified by the χ2 concordance test). The T-test was also used for one sample and the level of significance was set at α=0.05 (p<0.05).

### 2.5 Characteristics

The study enrolled 115 people, including 54 women (47%) and 61 men (53%) (p=0.144). All subjects qualified for the study and were operated on due to cancer metastasis to the axial skeleton based on the assumed selection criteria. The age of the subjects ranged from 36–86 years and the mean age ranged from 64.12 ± 10.60 years. The largest group (61.7%, n=71) was patients aged 61–86. More than half of the respondents (57.4%, n=66) were urban dwellers. Most of the respondents (79.1%, n=92) were married and people (63.9%, n=73) with secondary education dominated. Half of the respondents (50.4%, n=58) declared their socioeconomic status at the level of the national average (**Table 1**).

**Table 1:** Socio-demographic characteristics of the respondents.

Data categories		N	%
Sex	female	54	47.0%
	male	61	53.0%
Age	36-60 y.	44	38.3%
	61-86 y.	71	61.7%
Settlement	urban	66	57.4%
	rural	49	42.6%
Marital status	married	91	79.1%
	widower/widow	16	13.9%
	single	8	7.0%
	other	0	0.0%
Education	elementary	4	3.5%
	vocational	36	31.3%
	secondary	62	53.9%
	university	13	11.3%
Socioeconomic status	above the national average	11	9.6%
	national average	58	50.4%
	below the national average	46	40.0%

## 3 Results

### 3.1 Clinical and functional state of the studied patients

**Figure 2:**
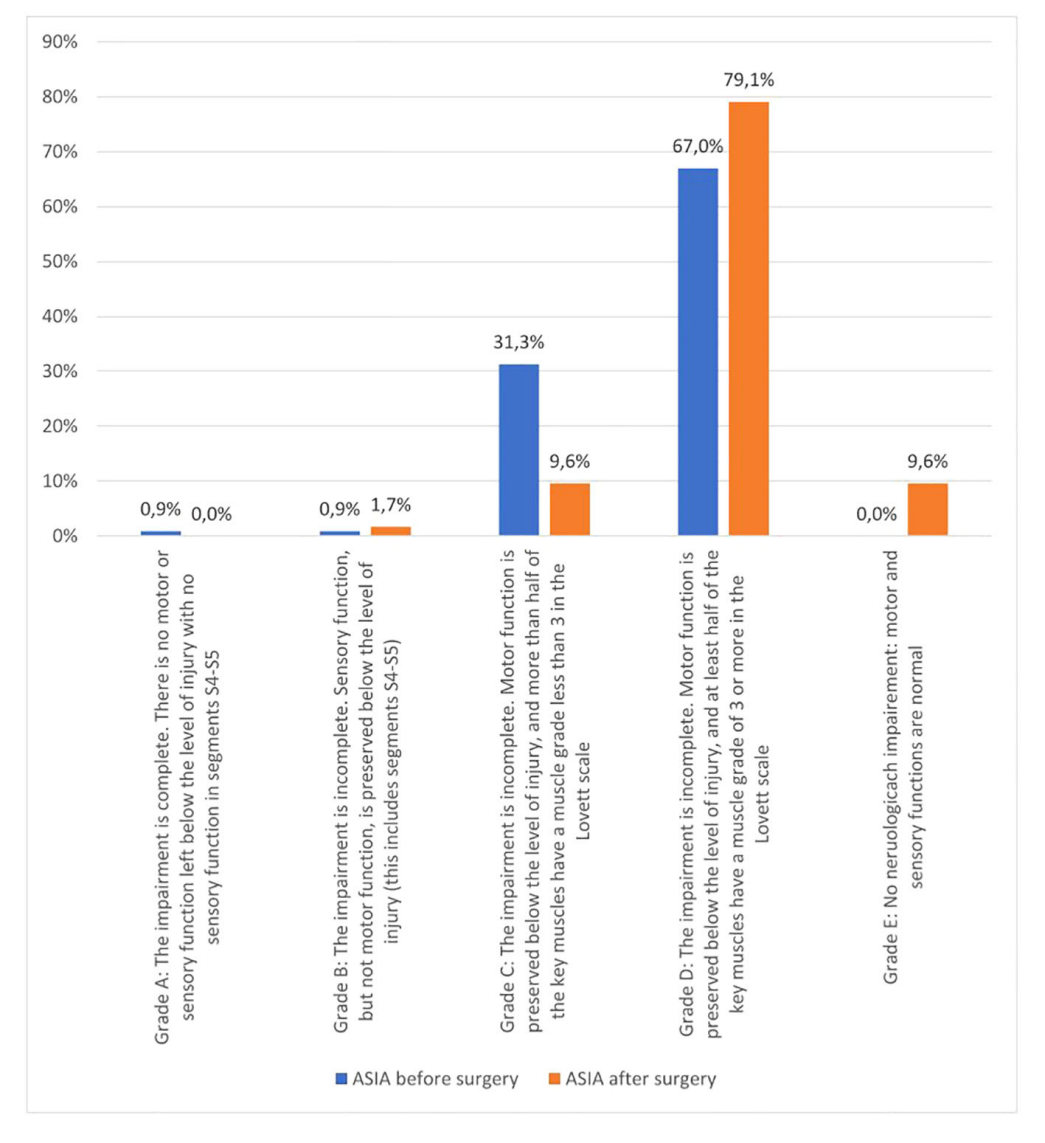
Clinical evaluation of the subjects before and after the surgery in ASIA.

More than half of the respondents (52.2%) had comorbidities including diabetes mellitus, neurological diseases and chronic obstructive pulmonary disease (COPD). The most common malignancies causing spinal metastases were breast cancer (27.0%) and multiple myeloma (27.7%), followed by prostate cancer (12.2%), lung cancer (10.4%) and kidney cancer (9.6%). The patients qualified for surgery due to decreased muscle strength within the girdles and the lower (61.7%) and upper (31.3%) limbs or pain (87.7%). The metastatic lesions within the spine were mainly located in the thoracic and lumbar sections. The clinical evaluation was based on the ASIA scale. In the pre-operative assessment, the majority of subjects (67.0%) presented with ASIA D neurological dysfunction (incomplete lesion: retained movement below the lesion level and more than half of the key muscles have strength equal to or greater than 3 in the Lovett scale). In the postoperative period, complete spinal damage was not found in the study group (grade A), an increase of 0.8% in grade B and 12.1% in grade D was observed, while a decrease of 21.7% in grade C. Normal motor and sensory activity (grade E) was found in 9.6% of the respondents compared to the preoperative period. The obtained data indicate an improvement in neurological functions after surgery in the ASIA scale (**Figure 2**).

**Figure 3:**
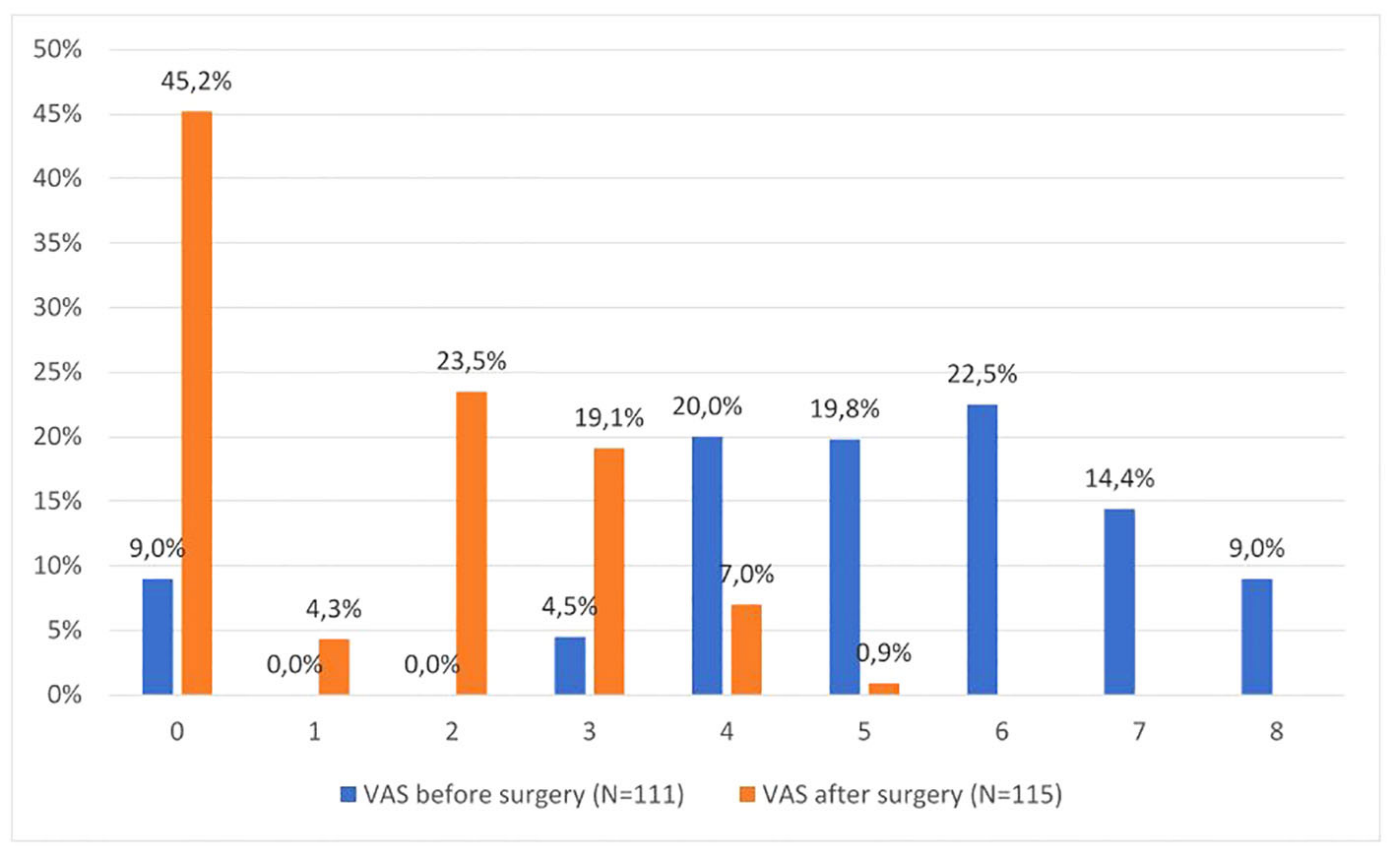
The level of pain in VAS perceived by the respondents before and after the surgery.

VAS pain perception before surgery was assessed in 111 people (i.e. 96.5% of the respondents). Four people were not able to reliably assess pain due to their health condition and because they were taking strong medications. Before the surgery, pain was experienced by 87.7% of the respondents, including more than half at the level of 6–8 points in VAS, 8.7% of the respondents did not report any pain. Before the surgery, the average level of perceived pain in the respondents was 5.04 ± 2.07 pts and ranged from 0 to 8 points, while postoperatively (in stage II of the study) it was 1.41 ± 1.44 pts and ranged from 0 to 5 pts (**Figure 3**). Pain severity after surgery decreased the quality of life of the respondents in terms of activity (p=0.025). The level of pain before and after the procedure did not influence other analysed variables related to the patients’ quality of life (p>0.05).

The activities of daily living in the study group were assessed using the ADL scale. In the preoperative period, the highest level of self-care was noted in terms of feeding (95.7%) and dressing (89.6%) and was lower in terms of bathing (45.2%) and continence (55.7%). After surgery, the best results were obtained in terms of feeding independently (97.4%) and dressing (93%) but were lower in continence (63.5%) and bathing (56.5%) (**Table 2**). ADL assessment revealed that 46.1% of the subjects were fully functional preoperatively, while this was 66.1% in the postoperative period (p<0.001). The number of people who were moderately disabled (40.9% before vs. 28.7% after) and significantly disabled (13.0% before vs. 5.2% after) decreased postoperatively.

**Table 2:** Independence of the respondents in performing activities of daily living in the pre- and postoperative period (ADL scale).

Self-care	Before surgery				After surgery			
	No		Yes		No		Yes	
	N	%	N	%	N	%	N	%
Bathing	63	54.8%	52	45.2%	50	43.5%	65	56.5%
Dressing	12	10.4%	103	89.6%	8	7.0%	107	93.0%
Toileting	41	35.7%	74	64.3%	20	17.4%	95	82.6%
Transferring	44	38.3%	71	61.7%	14	12.2%	101	87.8%
Feeding	5	4.3%	110	95.7%	3	2.6%	112	97.4%
Continence	51	44.3%	64	55.7%	42	36.5%	73	63.5%

### 3.2 Acceptance of the disease in the subjects

**Figure 4:**
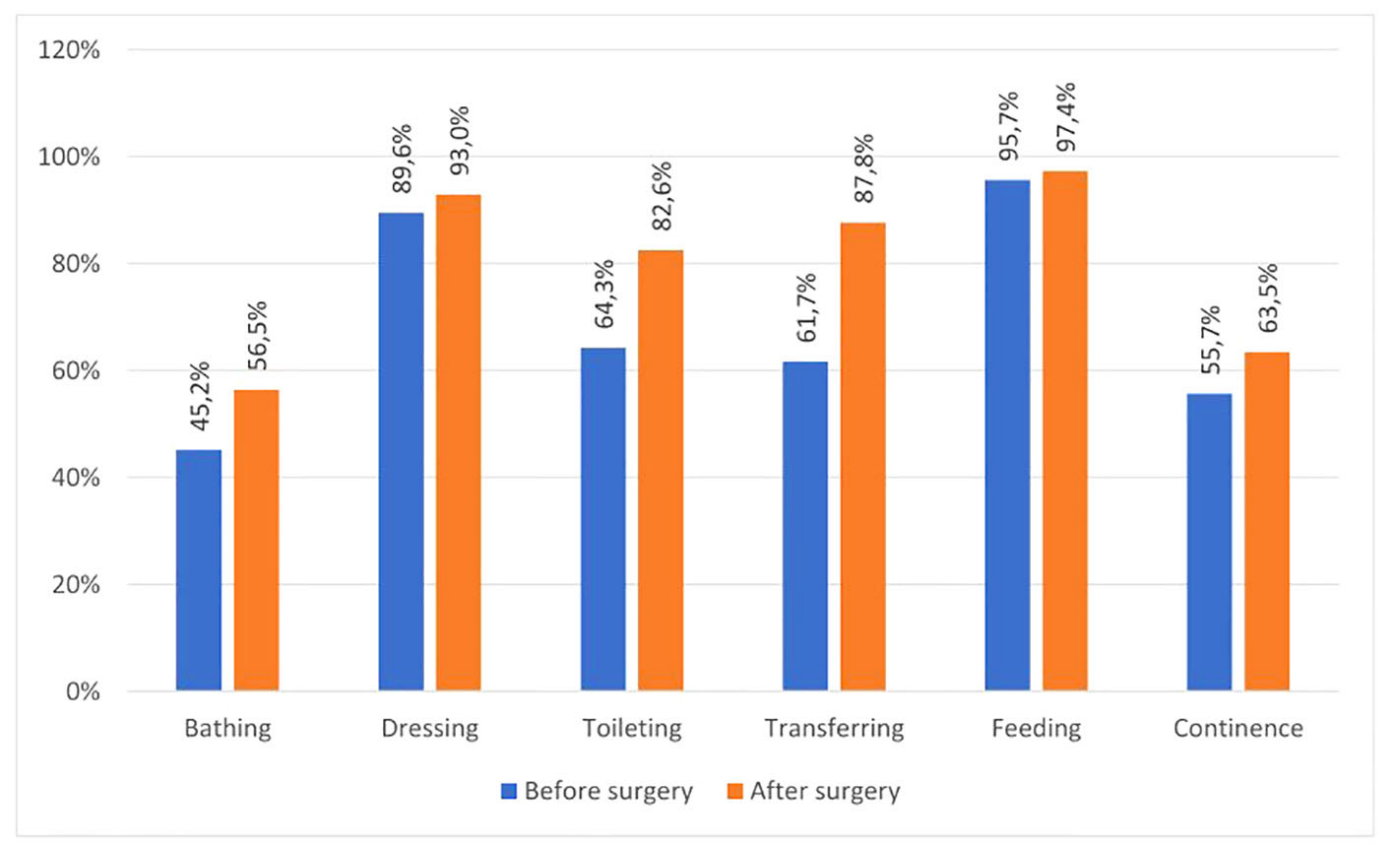
The level of disease acceptance in the study group (pre- and postoperatively).

The mean level of acceptance of the disease in the respondents before the surgery was 16.99 ± 6.65 points and ranged from 8–40 points. Half of the respondents achieved a result below 16 points. The mean result was lower (p<0.001) than the median value of the scale of 8–40 points, which was 24 points. The mean level of disease acceptance in the respondents after surgery was 22.66 ± 6.64 points and ranged from 8–36 points. Half of the respondents achieved a result below 23 points. The mean result was slightly lower (p=0.032) than the median value of the scale. The analysis demonstrated a higher level of disease acceptance in the respondents postoperatively than before the surgery (**Figure 4**).

### 3.3 Quality of life in the study group

The RSCL questionnaire was used to assess the quality of life of the respondents, while the general assessment of the quality of life encompassed the physical and psychological sphere and activity levels. The data assessing the quality of life obtained in RSCL were transformed into a score ranging from 0–100 points to enable objective comparison. The subjective assessment of the quality of life of the respondents after surgery was found to be significantly higher (p<0.001) than before it. Before the surgery, 57.4% of the respondents assessed the overall quality of life on average, while 41.7% as rather bad and bad. However, after surgery, 53.9% of the respondents assessed the overall quality of life at an average level and 40.0% rather good or good. The mean level of the overall quality of life of the respondents before the surgery was 44.4 and after the surgery it was 36.3. Before the surgery, the patients’ quality of life was lower in terms of physical symptoms (30.71 ± 11.96 points) and level of activity (36.56 ± 22.43 points). However, the level of psychological sphere after surgery was 31.35 ± 14.86 points. Statistical analysis showed a higher quality of life after surgery (p<0.001) in terms of the perception of physical symptoms (30.71 ± 11.96 points before the procedure vs. 20.91 ± 13.00 points after the procedure) and psychological symptoms (43.98 ± 14.82 points before surgery vs. 31.35 ± 14.86 points after surgery). The level of activity of the respondents also improved (p<0.001; 36.56 ± 22.43 points to 43.55 ± 20.40 points) (**Table 3**).

**Table 3:** Comparison of the descriptive statistics of the quality of life of the respondents in the analyzed spheres (pre- and postoperatively).

	Physical symptoms scale (before)	Physical symptoms scale (after)	Psychological symptoms scale (before)	Psychological symptoms scale (after)	Activity level scale (before)	Activity level scale (after)
Mean	30.71	20.91	43.98	31.35	36.56	43.55
SD	11.96	13.00	14.82	14.86	22.43	20.40
Min	2.90	0.00	9.52	0.00	0.00	0.00
Max	50.72	50.72	85.71	80.95	100.00	83.33
Q1	20.29	10.14	38.10	23.81	16.67	25.00
Q2 (Me)	34.78	18.84	42.86	33.33	37.50	45.83
Q3	39.13	34.78	52.38	42.86	58.33	62.50
p-Value	**<0.001**		**<0.001**		**<0.001**	

### 3.4 The respondents quality of life and selected variables (comorbidities, lesion sites, neurological disorders, muscle strength and the level of perceived pain)

Data related to the quality of life were compared with selected variables that may determine it. There was no correlation between gender, place of residence, marital status and quality of life. Younger people from the age group 36–60 years (50.57 ± 18.21) showed a higher level of activity after surgery than those aged 61–86 years (39.20 ± 20.59). The differences were statistically significant. A reduced quality of life in terms of the level of activity both before and after surgery (21.35 ± 22.87 vs. 24.48 ± 14.68) was observed in the group of patients diagnosed with diabetes (p=0.043 vs. p=0.006) and COPD (p=0.011 vs p=0.002). Regarding comorbidities, only diabetes and COPD had a negative impact on the quality of life in terms of activity. The site of neoplastic lesions was compared with the scales comprising the RSCL and no correlation was found between cervical and lumbar neoplastic lesions and the patients’ quality of life before and after surgery (p>0.05). The subjects with lesions localised in the thoracic spine presented a higher quality of life before the procedure in terms of level of activity (40.36 ± 23.26) compared to the group with lesions located in a different part of the spine (31.25 ± 20.29). The relationship between the patients’ quality of life before the surgery in terms of level of activity and thoracic metastases was confirmed (p=0.034). After surgical procedures, no statistical differences were found that conditioned the improvement of functionality (p>0.05). The site of the lesions in the sacral section decreased the quality of life of the patients before surgery in terms of psychological symptoms (p=0.025). After surgical procedures, no statistical differences were found that conditioned the improvement of functionality (p>0.05). No correlation (p>0.05) was found between the incidence of continence disorders and the subjective quality of life of the respondents before and after surgery.

The severity of pain after surgery decreased the quality of life of respondents in terms of activity (p=0.025). The level of pain before and after the procedure did not influence other analysed variables related to the patients’ quality of life (**Table 4**).

**Table 4:** The respondents’ quality of life (pre- and postoperatively) and the level of pain in VAS.

		Physical symptoms scale (before)	Psychological symptoms scale (before)	Activity level scale (before)
Pain on VAS scale [0-10 points] (before)	Rho	-0.108	-0.034	0.087
	p-Value	0.258	0.719	0.365
		Physical symptoms scale (after)	Psychological symptoms scale (after)	Activity level scale (after)
Pain on VAS scale [0-10 points] (after)	Rho	0.129	0.095	-0.208
	p-Value	0.170	0.313	**0.025**

The subjects who obtained a higher ASIA clinical score after surgery presented a better quality of life in terms of physical (p=0.002) and psychological symptoms (p=0.003). Similar observations were found in terms of activity. The subjects who presented a higher quality of life after surgery had fewer neurological dysfunctions (p<0.001) (**Table 5**).

**Table 5:** Descriptive statistics of the patients’ quality of life and clinical evaluation according to ASIA after surgery.

Clinical evaluation on ASIA scale		Physical symptoms scale (after)	Psychological symptoms scale (after)	Activity level scale (after)
A/B/C	Mean	25.53	40.66	23.72
	SD	14.77	18.61	18.35
	Me	18.84	33.33	20.83
	Min	10.14	19.05	0.00
	Max	50.72	80.95	66.67
	N	13	13	13
D	Mean	21.60	32.13	43.68
	SD	12.42	13.14	18.06
	Me	18.84	33.33	45.83
	Min	1.45	4.76	12.50
	Max	43.48	61.90	79.17
	N	91	91	91
E	Mean	9.75	13.85	65.91
	SD	10.29	9.63	18.52
	Me	4.35	9.52	70.83
	Min	0,00	0,00	20,83
	Max	34,78	33,33	83,33
	N	11	11	11
Total	Mean	20,91	31,35	43,55
	SD	13,00	14,86	20,40
	Me	18,84	33,33	45.83
	Min	0.00	0.00	0.00
	Max	50.72	80.95	83.33
	N	115	115	115
**p-Value**		**0.002**	**0.003**	**<0.001**

The quality of life of respondents, both before and after the procedure, was related to physical and psychological symptoms and level of activity; a higher intensity of physical and psychological symptoms and a lower level of activity determined low quality of life before and after the procedure. While analysing the subscales making up the RSCL, it was observed that the greater the intensity of physical symptoms (p=0.022) and psychological symptoms (p=0.017) and the lower the level of activity of the respondents (p<0.001), the lower the subjective assessment of their quality of life was before surgery. After surgery, those assessing the quality of life as rather good at the same time presented a higher quality of life in terms of physical symptoms (15.47 ± 11.79 points), psychological symptoms (23.81 ± 13.54 points) and a higher quality of life in terms of activity (53.62 ± 18.38 points). Lower results (p<0.001) in the areas of quality of life were presented by the respondents with an average/rather bad/bad subjective assessment of quality of life. Similar observations were made when analysing acceptance of the disease and the assessed quality of life. Before surgery, a higher level of disease acceptance corresponded to a higher level of quality of life related to the level of activity (p=0.004). After surgery, a higher level of disease acceptance determined a higher quality of life in terms of physical (p<0.001) and psychological symptoms (p<0.001) and a higher level of activity (p=0.004).

The level of independence in performing activities of daily living (according to ADL) differed significantly (p<0.001) compared to the preoperative period. After operations, the percentage of non-disabled people increased (66.1%) and the percentage of moderately disabled (40.9% before vs. 28.7% after) and significantly disabled people (13.0% before vs. 5.2% after) decreased. It was found that the level of functionality in performing ADL before the procedure influenced the quality of life in terms of physical symptoms (p=0.012), psychological symptoms (p=0.004) and activity (p<0.001). The subjects who were significantly disabled before the procedure had a reduced quality of life in terms of physical and psychological symptoms, while the quality of life associated with activity increased with improvements in functional status in terms of performing ADL. The independence of the respondents in performing ADL after surgery influenced the quality of life, both in the case of physical (p=0.006) and psychological symptoms (p=0.001) and activity (p<0.001). With the improvement of independence, the quality of life increased in the case of symptomatic scales and the level of activity increased.

## 4 Discussion

Extending the life expectancy of cancer patients inevitably leads to an increase in the number of complications associated with all systems and organs affected by the disease. Disorders related to the skeletal system (MESCC) determine motor problems and limitations in the mobility of joints, cause pain and reduce the efficiency of patients. Injury to the spine by metastatic lesions is a source of severe pain of biological origin, which mainly intensifies at night (18–20). Progressive bone destruction leads to the loss of spine stability and intensification of pain and increases the risk of pressure on the nervous structures leading to sensory disturbances, paresis and sphincter dysfunction (21). Bone metastases often cause pathological fractures that prevent the independent functioning of patients (11). Bedridden status results in an increased rate of complications in the form of deep bedsores, pneumonia and venous thrombosis of the lower extremities causing premature death. Numerous studies have shown that the use of surgical treatment had a positive effect on the life expectancy of patients and improved quality of life, both physically and mentally (9, 11, 19, 20). Patients with MESCC treated with the use of surgical methods aimed at decompression of the spinal cord followed by postoperative radiotherapy to retain their functional capacity for longer compared to the group of patients treated only with radiotherapy. According to Patchell et al., surgical intervention allows most patients to function at the outpatient level for the rest of their lives, while patients treated with radiation alone are much more likely to have severe neurological dysfunctions. The authors point out that surgical treatment increases the expected survival time (8).

The obtained results indicate that surgery within the axial skeleton (vertebroplasty, kyphoplasty) determines the improvement in respondents’ functionality and quality of life (p<0.001). Fewer neurological dysfunctions translated into a higher quality of life in terms of physical (p=0.002) and psychological symptoms (p=0.003). The independence of the respondents in activities of daily living (ADL) also improved, which determined a higher quality of life in terms of physical symptoms (p=0.006), psychological symptoms (p=0.001) and activity (p<0.001). Similar results were obtained by Barzilai et al., proving that the subjective and objective results of the treatment of patients with spinal metastases improved thanks to the implemented surgical treatment (22). High assessments (subjective and objective) of the quality of life prove the effectiveness of the undertaken surgical treatment. This observation is in line with the views of numerous authors dealing with the problem of metastatic tumours of the spine (11, 23–26).

Pain is the most studied symptom experienced by people with locomotor dysfunction in the course of cancer. Observational studies suggest that it is one of many disturbing symptoms that should be always minimised, especially in the advanced stages of disease (27). In the presented study, pain and neurological dysfunction were some of the most common negative symptoms reported by patients. A significant reduction in the level of pain was observed after surgery (mean before surgery 5.04 vs 1.41). Reducing pain predisposes to improved functionality and increases the activity of patients, thus improving the subjective assessment of the quality of life. Similar results were presented by Joubert et al. (28) and Chong et al. (29), who achieved pain reductions from 6.3 to 3.2 in VAS. According to Biega et al., corpectomy, like other surgical techniques, allows the pain associated with the compression of nerve trunks to be reduced effectively (30). Disease acceptance is one of the key measures of the adaptation process and is also one of the recognised predictors of survival. It gives a sense of security and reduces the intensity of negative reactions and emotions related to the disease itself and mental discomfort. Higher levels of disease acceptance predispose to lower levels of stress and higher self-esteem, which makes it easier to adapt to the disease. Our study showed that the subjective assessment of the quality of life is positively correlated with disease acceptance. After surgery, the respondents who had better acceptance of the disease (27.13 ± 4.76 points) presented a better self-assessment of the quality of life than those who accepted their disease to a lesser extent (19.68 ± 6.03). The level of disease acceptance in the study group was higher after the surgery compared to the assessment before the surgery. When analysing the literature, no studies on the acceptance of the disease in patients with spinal metastases were found; nevertheless, the above observations are in line with the results of studies carried out on groups of chronically ill patients.

Until recently, most measures of efficacy in treating spinal metastases focused on patient survival and relapse rates, complications, or function measures and neurological status. Less attention was paid to how patients characterise and describe their own health (24). Currently developed specialist palliative care offers great opportunities for improvements and the proper functioning of patients in advanced neoplastic disease if it is implemented as early as possible (31). In the public consciousness, cancer remains primarily a fatal disease, marked by physical and mental suffering, requiring long-term and often exhausting treatment. Patient-cantered care after spinal stabilisation requires a comprehensive collaborative teamwork of specialists in medicine and health sciences. The assessment of negative symptoms with questionnaires allows the directions of activities in comprehensive care aimed at identifying disturbing symptoms to be determined, as well as improving comfort and a better subjective quality of life.

### 4.1 Limitations

The study included patients treated in one centre in Poland, which does not allow the conclusions obtained to be generalised. A general assessment of the subjects’ quality of life was performed before the surgery and after 3–4 months, not taking into account the type of surgery performed and the long-term follow-up over 6 months. No analysis was performed comparing the type of procedure performed and other variables.

## 5 Conclusions

Studies have shown that spinal cord decompression surgery improves patients’ quality of life by reducing neurological dysfunction and increasing the acceptance of the disease and the possibility of self-care. The intensification of physical and mental symptoms as well as decreased activity is a destructive symptom for the respondents, reducing their subjective assessment of quality of life. Maintaining an optimal level of self-care predisposes to improved quality of life. Sociodemographic variables (before and after surgery) did not affect the respondents’ quality of life.

## Data availability statement

The original contributions presented in the study are included in the article/supplementary material. Further inquiries can be directed to the corresponding author.

## Ethics statement

The study was approved by the institutional Bioethics Committee at the University of Rzeszów (Resolution No. 2016/12/7; approved on 01-12-2016) and by all appropriate administrative bodies. The study was conducted in accordance with ethical standards laid down in an appropriate version of the Declaration of Helsinki and in Polish national regulations. Written informed consent for participation in this study was provided by the participants’ legal guardians/next of kin.

## Author contributions

Conceptualization, BB and DB. Methodology, BB, DB, and GG. Formal analysis, MK and PW. Investigation, BB and DB. Data curation, BB, DB and PW. Writing—original draft preparation, BB and DB. Writing—review and editing, BB, DB and PW. Supervision, GG, MK, RŚ and PW. All authors contributed to the article and approved the submitted version.
